# Capacitive Feedthroughs for Medical Implants

**DOI:** 10.3389/fnins.2016.00404

**Published:** 2016-09-08

**Authors:** Sven Grob, Peter A. Tass, Christian Hauptmann

**Affiliations:** ^1^Research Center Juelich, Institute of Neuroscience and Medicine 7 – NeuromodulationJuelich, Germany; ^2^Department of Neuromodulation, University of CologneCologne, Germany; ^3^Department of Neurosurgery, Stanford UniversityStanford, CA, USA

**Keywords:** electrical feedthrough, coupling capacitor, casing, capacitive feedthrough, additive manufacturing, medical implant, barium titanate

## Abstract

Important technological advances in the last decades paved the road to a great success story for electrically stimulating medical implants, including cochlear implants or implants for deep brain stimulation. However, there are still many challenges in reducing side effects and improving functionality and comfort for the patient. Two of the main challenges are the wish for smaller implants on one hand, and the demand for more stimulation channels on the other hand. But these two aims lead to a conflict of interests. This paper presents a novel design for an electrical feedthrough, the so called capacitive feedthrough, which allows both reducing the size, and increasing the number of included channels. Capacitive feedthroughs combine the functionality of a coupling capacitor and an electrical feedthrough within one and the same structure. The paper also discusses the progress and the challenges of the first produced demonstrators. The concept bears a high potential in improving current feedthrough technology, and could be applied on all kinds of electrical medical implants, even if its implementation might be challenging.

## Introduction

Neuromodulation has helped hundreds of thousands of patients so far, although it is a very young and still developing technology. Medical implants for neuromodulation apply small electrical current pulses on neural tissue to stimulate or inhibit nerve signals in different locations of the body. Deep Brain Stimulation (DBS), for example, stimulates specific regions in the brain to reduce or even prevent symptoms in Parkinson's disease, essential tremor, and other diseases (Coffey, 2009). Cochlear Implants help people with strong or total hearing loss to restore basic hearing capacities (Zeng, 2012). Until 2013, more than 100.000 implanted devices for DBS and over 300.000 cochlear implants have highly improved the quality of life of their recipients (Zeng, 2012; Roland and Tobey, 2013; McIntyre et al., 2014).

Neuromodulators consist of an implantable pulse generator (IPG), which contains the battery and the electronics. The IPG creates charge-balanced, biphasic stimulation signals. These signals are usually transmitted to the target tissue by electronic leads, which are connected to the IPG. In the electronics, one coupling capacitor is integrated in every stimulation channel to fulfill two major safety functions. At first, they are used to limit the maximum applied charge per pulse. Empirical studies derived a load density of 30 μC/cm^2^/phase as a maximum threshold for the charge density to avoid neural tissue damage by stimulation (Kuncel and Grill, 2004; Coffey, 2009). Furthermore, they prevent long-term charge-imbalanced stimulation, which could otherwise also result in serious tissue damage (Coffey, 2009; Hauptmann et al., 2009).

According to (Lee et al., 2013), one of the main future trends for deep brain stimulation will be the development of head mounted IPGs, which bear many advantages in comparison to present neurostimulators. Such developments are furthered by novel battery technology (Mallela et al., 2004) as well as novel stimulation protocols that, e.g., aim at inducing long-lasting effects, outlasting cessation of stimulation, and hence, delivering considerably less stimulation current (Tass and Majtanik, 2006; Tass et al., 2012; Adamchic et al., 2014). Surgery would be faster and cheaper as less operating sites would be necessary. Moreover, the risk of infection, which varies in literature from less than 1 to more than 15% (Hamani and Lozano, 2006; Benabid et al., 2009), would be reduced due to less lead extensions and especially a smaller implant surface. Moreover, the risk of lead fracture, which was also estimated to be within 1 and 15% (Hamani and Lozano, 2006), could be reduced as less and shorter leads and adaptors would be necessary. But, on the other hand, to allow a head mounted IPG the total implant size has to be drastically reduced (Coffey, 2009).

Furthermore, also increasing the number of stimulation contacts bears advantages for almost all neurological implants. This can be interesting, for example, to allow a selective picking of the most effective contacts after implantation or for reaching more neurons (Simeral et al., 2011; Hochberg et al., 2012; Wark et al., 2013). Another interesting idea is to use segmented electrodes, which allow a certain degree of steering of the electric field for a directed stimulation (Buhlmann et al., 2009, 2011; Contarino et al., 2014). The segmentation not only allows using different directions for the electrical field, but also to choose different stimulated volumes by enabling multiple different segmented contacts. This optimization of the stimulated area can improve therapy effects and reduce side effects at the same time. Common neurostimulators, like the Activa PC 37601 (Medtronic, Minneapolis, USA) and the Vercise DBS System (Boston Scientific, Valencia, USA), already use a total of 16 contacts on two leads. Sapiens Steering Brain Stimulation (Eindhoven, Netherlands) published an electrode containing even 32 contacts (Contarino et al., 2014). And equally in the field of cochlear implants systems with 20 electrodes are commonly used (Fishman et al., 1997). For each stimulation channel usually one coupling capacitor is used to prevent a resulting DC-current in case of electronic failure (Shepherd et al., 1999). At present mainly ceramic multilayer capacitors or capacitor arrays are integrated into the implants electronics, which occupy a significant volume of the electronics. With an increasing number of channels the coupling capacitors need even more space in the electronics. This prevents a highly demanded miniaturization of implants.

Besides the coupling capacitors each stimulation contact also needs a separate electrical feedthrough through the casing. It is very important that the feedthrough is hermetically sealed, especially against the entry of body fluids, which presents a high danger for the patient. Entering fluids could damage or destroy the electronics or even cause dysfunctions of the electronics. As this could not only lead to interruption of stimulation, but also to unwished malfunctioning it is very important to hermetically seal the implant (Donaldson, 1976; Jiang and Zhou, 2010). The common standard to build up a ceramic-to-metal feedthrough is by integrating platinum pins into isolative ceramic plates, e.g., from an alumina substrate, in a co-firing process (Zhou et al., 2007). These ceramic plates are embedded into a titanium frame, which can be laser-soldered to the titanium casing. This complex process needs many components, occupies a lot of space and limits the freedom of design for the implant. And nevertheless these feedthroughs still present a potential risk for the patient, as regularly problems with hermeticity occur (Thwaites, 1995). So it is another factor that limits or hardens miniaturization of the implant, and it also worsens with increasing number of stimulation channels (Bronstein et al., 2011; McIntyre et al., 2014). Implant size could be minimized, if the required space for coupling capacitors, and for electrical feedthroughs were reduced.

One way to minimize implants could be to increase the feedthrough density, for which some approaches can be found in literature: a ceramic casing made by High Temperature Co-fired Ceramics technology (HTCC) for example can already integrate 232 feedthroughs on a disk of 10 mm diameter (Ordonez et al., 2012). In another horizontal approach screen printing is used to build a hermetic implant casing with up to 360 sealed feedthroughs with a total size of 25 × 25 mm^2^ (Schuettler et al., 2010). Both ways show the vast possibilities of advancing ceramic technologies for medical implants, especially allowing the development of higher feedthrough densities and thus a smaller occupied space by the feedthroughs, whereas both approaches highly limit the design of the casing and particularly of the feedthroughs. For the first concept the feedthrough area is manufactured of two plane halves, which are bonded by co-firing, and then integrated into the rest of the casing. The second concept requires a ceramic plate with several coatings soldered to a metal lid with the feedthroughs in between the two parts. Furthermore, both approaches cannot limit or even minimize the required space for essential coupling capacitors, which is increasing with a higher number of electrical channels, and feedthroughs.

This paper presents a new concept of how to use ceramic technologies to not only increase feedthrough density but also to highly minimize the required space for coupling capacitors by integrating them into the wall of a ceramic casing. The concept is called capacitive feedthrough. A capacitive feedthrough is a capacitive link between the in- and the outside of the casing combining the functionality of the electrical feedthrough and the coupling capacitor in one and the same structure. By the use of capacitive feedthroughs, the electronics could be designed much smaller and feedthroughs could be designed with a much higher degree of freedom, e.g., by the use of ceramic 3D-printing technologies. First test structures were produced to demonstrate the concept. However, the results were not yet sufficient for a full proof-of-concept. They revealed that it is still challenging to implement the concept for medical implants, e.g., to determine the appropriate production method, especially regarding suitable, and biocompatible materials and process parameters.

### The concept of capacitive feedthroughs

Capacitive feedthroughs could help to minimize medical implants and allow much more freedom in designing them, even if the feasibility of the concept still has to be proven. This part explains what capacitive feedthroughs are, how they are structured and how their necessary parameters can be calculated.

A capacitive feedthrough fulfills both the functionalities of an electrical feedthrough and of a coupling capacitor. It builds up an electrical contact for AC signals between the in- and the outside of the casing, and it limits the maximum transmitted charge per pulse.

**Figure 1:**
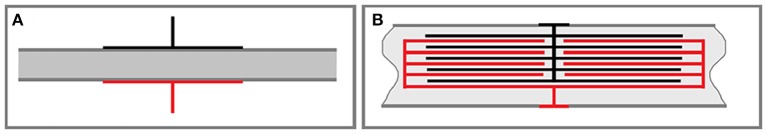
**(A)** Principle concept of a capacitive feedthrough: metal layers are added to the in- (red) and outside (black) of a non-conductive casing material (gray). The metal layers form a capacitive structure, which can be used to transmit charge pulses through the casing's wall, **(B)** advanced structure for a capacitive feedthrough: by integrating parts or all of the capacitive structure into the non-conductive material, dielectric widths can be decreased, and several capacitive layers can be added to greatly increase the resulting capacity.

For the capacitive feedthrough, the casing or at least a part of it must consist of a non-conductive material like a polymer or a ceramic. By adding conductive layers to both sides of the casing, a capacitive link over the casing wall is established, which is able to transmit biphasic stimulation signals to the other side. The conductive layers could be applied onto the wall (see Figure 1A) or even be integrated into the wall of the casing (see Figure 1B). The hermeticity of the casing is not affected by the additional layers. As a result, integration of the coupling capacitors into the wall without increasing its thickness reduces the necessary inner volume of the implant, as the coupling capacitor in the electronics is no longer necessary. Moreover, the complex and design limiting state of the art of electrical feedthroughs can be avoided.

The most suitable material for the concept seems to be barium titanate, as it shows the highest relative permittivity and also a great biocompatibility in *in-vitro* (Park et al., 1977; Beloti et al., 2006; Zhang et al., 2014) and *in-vivo* studies (Park et al., 1977, 1981; Lopes et al., 2013). Also conceivable would be the use of titanium dioxide, which has a well-known and well-tested biocompatibility, and a relative permittivity of around 110. Most other ceramics and polymers only show relative permittivities lower than ten, and would therefore not be well-suited for the concept (Lin and Yen, 2006; Kurzweil et al., 2008).

To calculate the capacity according to the concept (a), the equation for a standard plate capacitor can be applied (${\text{C}}_{\text{a}}=\varepsilon_{0}*\varepsilon_{\text{r}}*\frac{\text{A}}{\text{d}}$). For the first concept it is very important to produce the dielectric as thin as possible to reach a high capacity of the capacitive feedthrough, but on the other hand as thick as necessary to assure mechanical stability. Even with the optimistic assumption of a minimum thickness of d = 300μm, the use of barium titanate with a relative permittivity of ε_r_ = 9000 and the dielectric constant of $\varepsilon_{0}=8.85*10^{-12}\frac{As}{Vm}$, a common capacity of *C* = 470nF could only be produced with an electrode surface area of A = 1.8*10^3^ mm^2^. This is far too big to be competitive to the state of the art.

In contrast, by integrating multiple metal layers into the wall of the casing, the functional dielectric thickness between the two capacitor electrodes can be drastically reduced (see Figure 1B). The Capacity is multiplied by the number of neighboring bipolar metal plates ($C_{b}=\varepsilon_{0}\;*\;\varepsilon_{\text{r}}\;*\;\frac{{\text{A}}_{0}\;*\;\left(\text{N}-1\right)}{\text{d}})$. In this way the mechanical stability will not be reduced, presuming that the metal, and the ceramic layers form a strong mechanically robust compound. Industrial capacitor production already handle barium titanate layers of a thickness of less than d = 3μm for the dielectric layers (Friedrich, 2004). A capacitive feedthrough with such dielectric's and metal layer's thickness and with N = 20 integrated metal layers in a similar structure to Figure 1B would only need a surface area of A_0_ = 0.9 mm^2^ per plate. The height of the plate bundle would be around 120 μm, so that an exemplary thickness of an implant casing of 400 μm would be sufficient to totally integrate the capacitive feedthrough.

## Materials and methods

In order to produce a first demonstrator of the concept, two sample structures were designed, and produced in cooperation with the company WZR Ceramic Solutions GmbH (Rheinbach, Germany). The goal was to produce a simple structure containing capacitive feedthroughs and hence demonstrate the idea of the concept and its production method. In this chapter the production method with its materials and the design of the test structures are explained:

### Multi-material 3d-printing process

**Figure 2:**
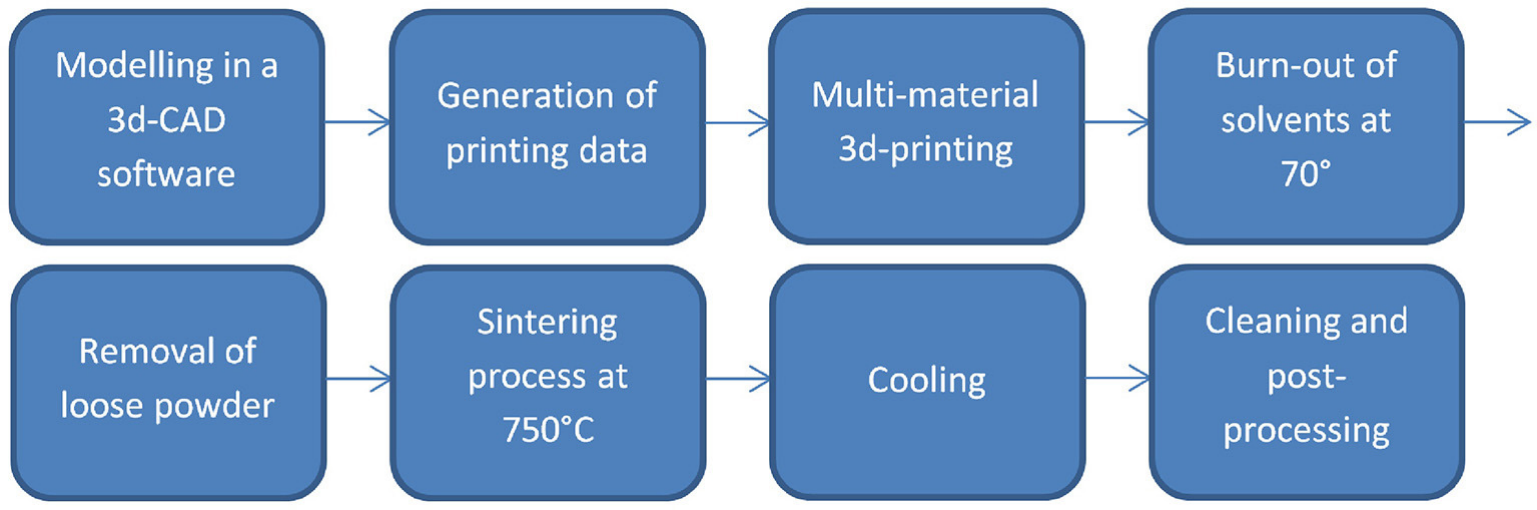
**Stages of the multi-material 3d-printing process**.

A layer based additive manufacturing technology called multi-material 3d-printing was used to produce the structured metal ceramic compounds. Figure 2 shows an overview of the stages of the process. It is based on a 3d model from a CAD system, which is sliced into single layers of 100 μm. In the production process, these 100 μm thick layers were formed consecutively by printing two different binder fluids on a ceramic powder layer. After printing one layer, a new layer of ceramic powder was added upon the yet printed layer. When all layers were finished, the solvents of the binder fluids were burned out at elevated temperatures for several hours before co-sintering the whole structure at 750°C for several hours. The patent protected process used special binder liquids containing metal particles to be able to add conductivity to certain structures or ceramic particles to increase density of the green body, which reduces shrinkage during sintering (Kollenberg, 2012, 2014). The right sintering temperature profile and the correct concentration and amount of silver binder were the most sensitive aspects of the process (Yan and Gu, 1996; Baumann, 2010). The goal was to receive a conductive structure within one layer, which would still be well-isolated to the other conductive layers nearby.

The applied printing process was in the time of the production still in a development phase and especially the process parameters were not yet optimized nor standardized.

### Used materials

At the time of the production, the company could only process a material combination of a glass-ceramic and silver in the multi-material 3d-printing process.

The silver particles had a size of less than 1 μm and were evenly distributed in the polymeric binder liquid used for printing of the conductive structures.

The glass ceramic was used as the ceramic powder basis with particle sizes of 1–80 μm. It mainly consisted of SiO_2_, which only has a permittivity of about 3.5. This was hence not the most suitable material. But as the focus was to proof the feasibility of the concept, and not to receive a very high capacity, the glass-ceramic material was still used for the two demonstrators. For a later functional prototype barium titanate should be used in combination with a suitable conductive material.

### Design

**Figure 3:**
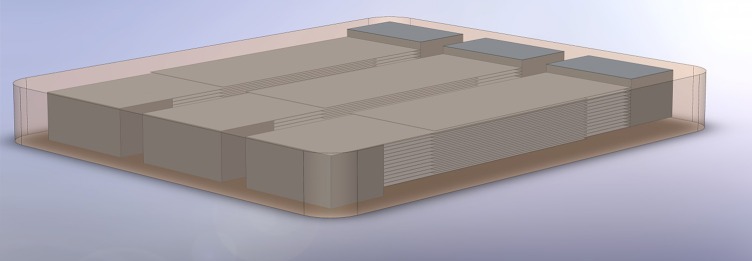
**Design of the second prototype: a glass-ceramic material with three integrated silver capacitors**. The glass-ceramic is shown semi-transparent in the graphic to reveal the inner silver structure. The structure consists of a total of 35 ceramic layers of about 100 μm and 26 integrated conductive silver layers for each capacitor. Each surface electrode is connected to half of the silver plates to form a capacitor electrode.

Figure 3 shows the design of a glass-ceramic part with three integrated capacitive structures made from silver. The design was used for the second prototype and a similar, but thinner and wider one for the first prototype. The expected calculated capacity of the three capacitors was only 0.85 nF each. With the low permittivity of the glass ceramic it was not possible to reach capacities of 100 nF and more, as it is common for coupling capacitors. The target dimensions were 33 × 28 × 3.5 mm, with an assumed shrinkage rate of 25%, which is based on the experience of the manufacturers.

### Measurement devices

For the electrical measurements a digital oscilloscope TDS 2024B from Tektronix (Beaverton, OR USA) together with the oscilloscope probe C3000 from Conrad Elektronics (Hirschau, Germany) was used.

## Results

Two demonstrators of the concept were produced in a multi-material 3d-printing process. The quality of the structures has been analyzed in a scanning electron microscopy and by electrical measurements of the conductivities and capacities of the integrated capacitive feedthroughs.

**Figure 4:**
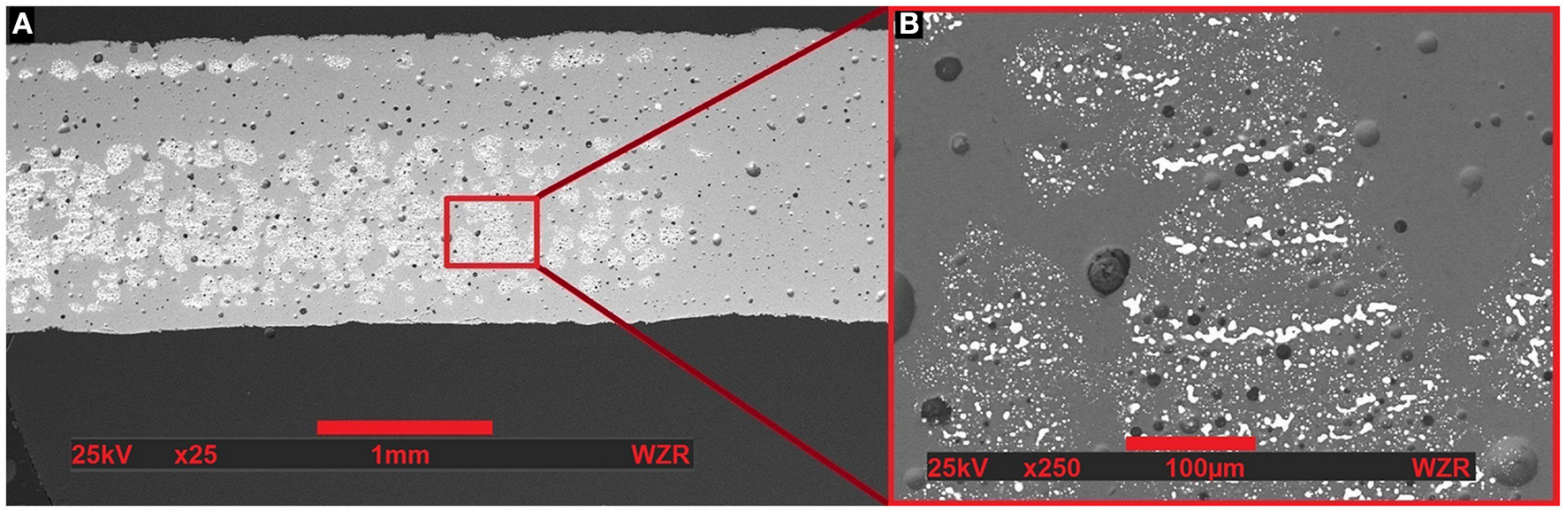
**Scanning Electron Microscopy of a cross-section of the first prototype with a magnification level of 25 (A) and 250 (B)**. The structure is upside-down. There are many pores in the glass-ceramic material (gray). However, the silver particles (white), and the glass-ceramic material formed a fully merged composite without pores at the interfaces. Many regions within one layer remained without silver, assumingly because the printed silver binder was not spread out homogenously. Furthermore the concentration of silver in the binder was not sufficient to build up a closed layer.

The first printed sample did not show any capacitive functionality, yet. Scanning Electron Microscopy revealed that the amount of silver binder droplets as well as the silver concentration in the binder itself were not sufficient (see Figure 4). But the process did result in a strongly bonded multi-material compound, which shows a fully merged structure without cracks in between the two materials. The general high amount of pores in the ceramics results from the omitted step of burning-out of the binder liquids in the first tests.

**Figure 5:**
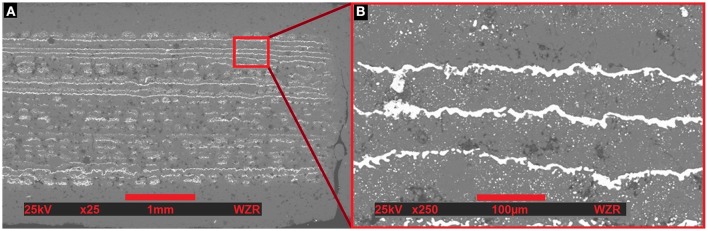
**Scanning Electron Microscopy of a cross-section of the second prototype with a magnification level of 25 (A) and 250 (B)**. Here the better division of silver binder and the higher silver concentration in the binder lead to some almost continuously formed layers, whereas other layers still show many gaps. Another result is that some elements of two different layers come pretty close to each other (e.g., picture **B** at the upper left corner). This should significantly decrease the breakdown voltage, or even could cause a direct shortcut.

As a consequence of the first printing test, the silver concentration in the binder, and the amount of silver droplets were increased for the second printed sample. All three capacitors of the second product were functional. However, the capacities only reached about 10% of the expected 0.85 nF. Moreover, two out of the three capacitors from the second sample showed a measureable DC resistance of several MΩ, whereas a capacitor should feature a much higher ideally infinite resistance. Scanning Electron Microscopy was likewise used to analyze the structure of the second sample (see Figure 5). The images manifest that the higher silver concentrations helped to form conductive layers of silver. Most of the upper layers in image (a) are totally formed, whereas in the lower section many layers still received insufficient silver droplets. After all, there are many spots in the layers without any silver at all. On the contrary, in image (b) in the upper left corner a protrusion of the upper silver layer reaches critically close to the next lower level. Assumingly, in this manner established contacts between two layers could be the reason for the low DC resistances.

The few fully established layers seem to be responsible for the measureable capacity. On the other hand, the many incomplete layers explain, why the capacity only reaches about 10% of the target capacity. The raised concentrations improved the formation of fully conductive layers, but nevertheless the amount of silver binder droplets is not sufficient and especially not homogeneously distributed well. Yet, also the best formed layers in the image still don't feature a fully continuous layer, but at the same time already present nearly interlinked sections in between two layers. And last but not least, exceeding mechanical stress due to inhomogeneous thermal expansion or shrinkage lead to several cracks in the ceramic part (see Figure 5A).

To test the functionality of the capacitors of the second print, a test signal was transmitted over the capacitors. DBS implant electronics (Hauptmann et al., 2009, modified electronics) were used to generate a biphasic, charge-balanced, current-driven stimulation signal. Amplitude of the signal was 0.1 mA, the frequency was 130 Hz, and the pulse width of the first pulse was 60 μs. The second, negative pulse only had a 10 of the amplitude, but showed a pulse width of 600 μs. To increase the functional capacity to approximately 170 pF, the three capacitors were connected in parallel. As this capacity is still much smaller than common capacitors of 470 nF, a strong current divider was used to strongly reduce the current through the capacitors by a factor of 1000. We measured the voltage of the transmitted test signal at a resistance of 1 kΩ with the oscilloscope described in chapter 3. The measurement was compared to the set signal recorded while using an industrial capacitor of 470 nF, instead.

**Figure 6:**
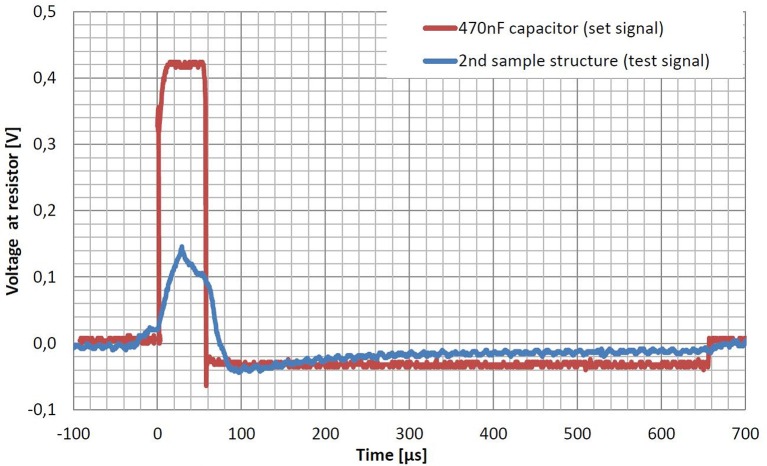
**Voltage of the transmitted stimulation signals using the three parallel capacitors of the second test structure (test signal) compared to the one using a common 470 nF capacitor (set signal)**. DBS implant electronics were connected in series to one of the two capacitors and a resistor of 1 kΩ simulating the tissue resistance. Stimulation parameters were a frequency of 130 Hz, a current of 0.1 mA and a pulse width of 60 μs for the positive pulse followed by a negative pulse of 600 μs and a tenth of the current. The extremely small capacity of about 170 pF of the sample structure made it necessary to use a current divider with a factor of 1000 to lower the current through the resistance and the capacitors. Both graphs show averages over 128 samples to reduce noise. The amplitude of the test signal only reaches 34% of the one from the set signal. Moreover the shape is strongly deformed due to saturation effects of the capacity, which is too small. A possible shift between the two graphs is due to different trigger options.

Figure 6 shows the measured signals using a 470 nF capacitor (set signal) and the three parallel capacitors of the second sample structure (test signal). The biphasic signal could successfully be transmitted through the three parallel capacitors. Nevertheless, the signal only reaches 34% of the set signal's amplitude. Both phases can be recognized, whereas they both result strongly deformed due to saturation effects of the too small capacity.

## Discussion

Common electrical feedthroughs limit the freedom in designing medical implants, especially as, as a general tendency, the number of stimulation channels, and electrical feedthroughs is increasing (Contarino et al., 2014; McIntyre et al., 2014). Furthermore, the resulting need for more coupling capacitors limits the highly demanded miniaturization of medical implants (Sit and Sarpeshkar, 2007; Jiang and Zhou, 2010). This paper presented a new concept called capacitive feedthrough, which could provide an option to overcome these challenges. However, the production of a casing implementing the concept seems challenging, as appropriate materials and an appropriate production method still have to be determined.

The biggest necessary change to implement the concept in medical implants is the use of a non-conductive casing material for the whole casing or at least a part of it. Biocompatibility, hermeticity, and relative permittivity are the main criteria for a suitable material. Especially the relative permittivity leads to a high limitation of qualified materials, so that the ceramics titanium dioxide and barium titanate seem to be the only materials, with which required capacities could be reached.

With advances in 3d-printing technology, thinner processable layer thicknesses could support the suitability of other materials as well. Especially the further development of impermeable polymers could be attractive, as they can already be processed in layer thicknesses much lower than 1 μm and still show a satisfying breakdown voltage.

So far, two first test structures containing capacitive feedthroughs have been produced. The first resulted in a strong metal-ceramic compound, but did not show capacitive functionality due to insufficient silver concentrations. However, the second one featured measureable capacities and allowed the transmission of a test signal, but on the other hand developed cracks during sintering. Summing this up, the parameters for designing, printing, and sintering capacitive feedthroughs still have to be largely improved to receive a fully functional and mechanically robust prototype. And even more important, other biocompatible materials for producing the capacitive feedthroughs, like barium titanate, have to be involved to allow a design reaching the common capacity of 470 nF.

Liu et al. also presented a way to reduce the necessary capacitor size by high-frequency current switching together with a bridge rectifier circuit (Liu et al., 2007), which possibly reduces necessary capacity by a factor of 20,000. This might be a very promising approach, particularly as it allows an integration of the coupling capacitor into the output stage, which could save space in the electronics. However, necessary fast switching times limit the effectivity of this concept, and moreover especially for higher voltage outputs fast switching implies higher power consumption (Sooksood et al., 2009).

Another approach to minimize implants is not to use coupling capacitors at all, but instead use alternative passive, or active charge balancing techniques. On one hand, passive charge balancing is performed by regular electrode short circuiting (Sit and Sarpeshkar, 2007; Sooksood et al., 2009). On the other hand, multiple approaches for an active charge balancing have been presented, e.g., by pulse insertion (Ortmanns et al., 2007) or offset regulation technique (Schuettler et al., 2008; Sooksood et al., 2010). Both the alternative passive and active techniques can provide a charge-balanced stimulation in standard circumstances, although the functionality of the passive approach is limited for higher stimulation frequencies. And finally both concepts have the serious disadvantage that in case of an IC-failure, they cannot, unlike a coupling capacitor, safely prevent DC-currents (Sooksood et al., 2009).

Although there are few alternative approaches, apparently in the near future the use of coupling capacitors for charge balancing and IC-failure safety will still be necessary (Sooksood et al., 2009). The concept presented in this paper might help to significantly reduce capacitor size in the future. This and the fact that the concept allows a high level of freedom in designing and positioning electrical feedthroughs could support the progression of head mounted implants, which bear many advantages in comparison to common chest-implanted brain stimulators (Lee et al., 2013). Major advantages might be a faster and cheaper surgery, a lower risk of infection, shorter, and less necessary lead extensions and a lower risk for lead fractures (Lee et al., 2013). However, there is still much work to be done in the development and certification of an implantable casing containing capacitive feedthroughs.

## Conclusion

The paper presented the new concept of capacitive feedthroughs. With the use of the concept medical implants might be safer (no perforations of the casing required, lower risk of infection), smaller (the size of feedthroughs decreases and no coupling capacitors are required on the printed circuit board), with more stimulation channels (less space for feedthrough means more feedthroughs possible, depending on size of capacitive structure), and with more freedom in design (3d-printing process allows high level of freedom).

However, it is to be said that it is still a big challenge to determine a suitable production method, especially regarding a material mix that combines biocompatibility, durability, lifetime, and appropriate electrical properties. Important aspects yet to consider are also the determination of a possible noise pickup by the conductive structures, which must be minimized, or prevented, and the minimum safe charge injection limits for the design. As a consequence of this, it is not yet sure how big the benefits can be in comparison to the common feedthrough and coupling capacitor designs and if the concept can actually be realized as proposed by this concept.

Despite the fact that capacitive feedthroughs still lack optimization, the use of the concept offers a high potential to overcome some major challenges in medical implants.

## Author contributions

The paper was written by SG based on the master thesis of SG. The project of the master thesis was supervised by PT and CH. The idea for the project was developed by CH. The paper was reviewed by PT and CH.

### Conflict of interest statement

Full financial disclosure for the previous 12 months: CH is employed by Juelich Research Center. He also works as consultant for Brook Henderson Group (DESYNCRA Technologies Limited), and has received research funding from the European Community, the Federal Ministry of Education and Research (Germany), the Deutsche Forschungsgemeinschaft, the Helmholtz Association. PT has a Director appointment at the Juelich Research Center and a faculty appointment at Stanford University. He has received research funding from the European Community, the Federal Ministry of Education and Research (Germany), the Deutsche Forschungsgemeinschaft, the Helmholtz Association, Biomedical Primate, the Michael J Fox Foundation. Two patents for the capacitive feedthrough technology have been applied for. The remaining author declares that the research was conducted in the absence of any commercial or financial relationships that could be construed as a potential conflict of interest.
